# Brain barriers virtual: an interim solution or future opportunity?

**DOI:** 10.1186/s12987-022-00314-2

**Published:** 2022-03-01

**Authors:** Brianna M. Holder, Shaina E. Tolan, Kaleb K. Heinrich, Kaitlin C. Miller, Natalie Hudson, Geetika Nehra, Michelle E. Pizzo, Steffen E. Storck, William F. Elmquist, Britta Engelhardt, Irena Loryan, Michal Toborek, Bjoern Bauer, Anika M. S. Hartz, Brandon J. Kim

**Affiliations:** 1 Department of Biological Sciences, Science and Engineering Complex, The University of Alabama, 300 Hackberry Ln, Tuscaloosa, AL 35487 United States; 2 Department of Journalism and Creative Media, The University of Alabama, Tuscaloosa, AL United States; 3 Neurovascular Genetics Unit, Smurfit Institute of Genetics, Trinity College Dublin, Dublin, Ireland; 4 Department of Biology, The University of Kentucky, Lexington, KY USA; 5 Denali Therapeutics Inc., CA South San Francisco, United States; 6 Washington University of St. Louis, St. Louis, MO USA; 7 Gutenberg Research Fellowship Group of Neuroimmunology, Focus Program Translational Neuroscience (FTN) and Immunotherapy (FZI), Rhine Main Neuroscience Network (rmn), University Medical Center of the Johannes Gutenberg University Mainz, Mainz, Germany; 8 Department of Pharmaceutics, College of Pharmacy, University of Minnesota, Minneapolis, MN United States; 9 Theodor Kocher Institute, University of Bern, Bern, Switzerland; 10 Translational PK/PD Group, Department of Pharmacy, Uppsala University, Uppsala, Sweden; 11 Department of Biochemistry and Molecular Biology, University of Miami Miller School of Medicine, Miami, FL USA; 12 Department of Pharmaceutical Sciences, College of Pharmacy, University of Kentucky, Lexington, Kentucky USA; 13 Sanders-Brown Center on Aging, University of Kentucky, Lexington, Kentucky USA; 14 Department of Pharmacology and Nutritional Sciences, University of Kentucky, KY Lexington, USA; 15 Department of Microbiology, Heersink School of Medicine, University of Alabama at Birmingham, Birmingham, AL USA

**Keywords:** Blood–brain barrier, BBV2020, Brain barriers virtual, Virtual seminar series, COVID-19, Education

## Abstract

**Background:**

Scientific conferences are vital communication events for scientists in academia, industry, and government agencies. In the brain barriers research field, several international conferences exist that allow researchers to present data, share knowledge, and discuss novel ideas and concepts. These meetings are critical platforms for researchers to connect and exchange breakthrough findings on a regular basis. Due to the worldwide COVID-19 pandemic, all in-person meetings were canceled in 2020. In response, we launched the Brain Barriers Virtual 2020 (BBV2020) seminar series, the first stand-in virtual event for the brain barriers field, to offer scientists a virtual platform to present their work. Here we report the aggregate attendance information on two in-person meetings compared with BBV2020 and comment on the utility of the virtual platform.

**Methods:**

The BBV2020 seminar series was hosted on a Zoom webinar platform and was free of cost for participants. Using registration- and Zoom-based data from the BBV2020 virtual seminar series and survey data collected from BBV2020 participants, we analyzed attendance trends, global reach, participation based on career stage, and engagement of BBV2020. We compared these data with those from two previous in-person conferences, a BBB meeting held in 2018 and CVB 2019.

**Results:**

We found that BBV2020 seminar participation steadily decreased over the course of the series. In contrast, live participation was consistently above 100 attendees and recording views were above 200 views per seminar. We also found that participants valued BBV2020 as a supplement during the COVID-19 pandemic in 2020. Based on one post-BBV2020 survey, the majority of participants indicated that they would prefer in-person meetings but would welcome a virtual component to future in-person meetings. Compared to in-person meetings, BBV2020 enabled participation from a broad range of career stages and was attended by scientists in academic, industry, and government agencies from a wide range of countries worldwide.

**Conclusions:**

Our findings suggest that a virtual event such as the BBV2020 seminar series provides easy access to science for researchers across all career stages around the globe. However, we recognize that limitations exist. Regardless, such a virtual event could be a valuable tool for the brain barriers community to reach and engage scientists worldwide to further grow the brain barriers research field in the future.

**Supplementary Information:**

The online version contains supplementary material available at 10.1186/s12987-022-00314-2.

## Introduction

On March 11, 2020, the World Health Organization (WHO) declared the coronavirus disease (COVID-19) outbreak a global pandemic [1]. Shortly after, countries worldwide issued stay-at-home orders and lockdowns: the COVID-19 pandemic had forced the world to come to an abrupt halt [2]. With global air travel restrictions and travel bans in place, enterprises including academic institutions, industry and government agencies required individuals to call off their business travel. As a result, scheduled scientific conferences were canceled throughout 2020 [3, 4]. These cancelations created a sudden void in scientific communication between researchers worldwide and forced research communities to be creative and adapt. One possibility to continue scientific communication and interaction was by switching conferences and seminars to virtual online events using novel digital platforms enabling researchers to connect and share science [3, 4].

As a consequence, major conferences and meetings in the brain barriers research field, including the “Barriers of the CNS Gordon Research Conference” (GRC 2020) and the international symposium on “Signal Transduction at the Blood–Brain Barriers” in Bari, Italy were first postponed until 2021 and due to the ongoing pandemic subsequently moved to 2022. The “Cerebral Vascular Biology” (CVB) conference in Uppsala, Sweden planned for 2021 was postponed until 2023. Postponing these meetings left organizers and conference chairs in a difficult position and researchers without a platform to present and share their data. Thus, these postponements created a critical need for the field to stay connected and an opportunity to establish a virtual event that could supplement the postponed conferences in 2020. The Brain Barriers Virtual 2020 Seminar Series (BBV2020) addressed this need by providing a forum for brain barrier scientists worldwide to come together online during the COVID-19 crisis in 2020. BBV2020 seminars were held weekly from May 20, 2020 to September 2, 2020 in a 1-h format featuring a live session with an invited speaker followed by time for questions and answers from the community. The seminar series was hosted on a Zoom webinar platform and was free for anyone to attend. BBV2020 served as a forum for invited speakers to present their work to the brain barriers community and enabled this community to discuss brain barriers science from the safety of their home office. Over 1300 scientists around the world were registered, each seminar was viewed live by 100–400 participants, and the video-recordings received 150–900 views each week. Participants were from academia, industry and government agencies at all career stages from around the globe.

In this report, we share participation data that give insight into the impact this virtual seminar series had on the brain barriers field during the COVID-19 pandemic. We compare these data with existing data from past in-person brain barriers meetings and draw conclusions from these data on the potential value of virtual meetings for the brain barriers research field in the future. While the organization of virtual events during the pandemic may seem self-evident, reflection on the impact carries utility for events in the future.

## Methods

### Organization

The BBV2020 seminar series was created by Drs. Bjoern Bauer (University of Kentucky, Lexington, KY, USA), Anika Hartz (University of Kentucky, Lexington, KY, USA), and Brandon Kim (University of Alabama, Tuscaloosa, Alabama, USA). Seminar moderators were postdoctoral researchers and scientists from the USA and Europe: Drs. Natalie Hudson (Trinity College Dublin, Dublin, Ireland), Geetika Nehra (University of Kentucky, Lexington, KY, USA), Michelle Pizzo (Denali Therapeutics Inc., San Francisco, CA, USA), and Steffen Storck (University Medical Center of the Johannes Gutenberg University Mainz, Mainz, Germany).

### Virtual platform and technical assistance

BBV2020 was run on the Zoom Webinar 500 platform (Zoom Video Communications, Inc.; San Jose, CA, USA) that was funded by Dr. Bjoern Bauer using University of Kentucky funds. This particular Zoom Webinar license allowed for up to 500 live participants. The Zoom Webinar platform supports an in-session chat function, a Q&A forum, a raise hand function for live questions, and provides a panelist section with the option to share audio, video and presentation slides, a recording function and post-webinar data collection. The hosts and moderators were in control of audio and video; participants were not able to record or unmute during a session. Participants had no control function other than a “raise hand” option and the Q&A and chat function allowing to send questions or messages to the moderator. Technical assistance was provided by Todd Sizemore, College of Pharmacy, University of Kentucky.

### BBV2020 schedule

The BBV2020 seminar series ran from May 20 to September 2, 2020. Live seminars were held once per week from 12 to 1 PM Eastern Daylight Time (EDT) to accommodate researchers across a wide range of time zones: Central European Time (6:00 PM) to Pacific Daylight Time (9:00 AM). To accommodate colleagues with time constraints or those within incompatible time zones such as Asia or Oceania, presentations were recorded (depending on speaker permissions) and the seminar recording was made available for up to 48 h after the live seminar. The link for the recorded videos was distributed via the BBV2020 listserv that was set up through the University of Kentucky listserv.

### Invited speakers

Efforts were made to invite speakers from a variety of countries and genders. Invited speakers included organizers of postponed in-person conferences, speakers from academia and industry from different career stages. A total of 16 speakers were invited: May 20, 2020: Elga De Vries, Amsterdam UMC, Amsterdam, The Netherlands; May 27, 2020: Richard Daneman, University of California San Diego, San Diego, CA, USA; June 3, 2020: Robyn Klein, Washington University, St. Louis, MO, USA; June 10, 2020: Ayal Ben-Zvi, The Hebrew University of Jerusalem, Israel; June 17, 2020: Britta Engelhardt, University of Bern, Bern, Switzerland; June 24, 2020: Margareta Hammarlund-Udenaes, Uppsala University, Uppsala, Sweden; July 1, 2020: Daniela Virgintino, University of Bari, Bari, Italy; July 8, 2020: Eric Shusta, University of Wisconsin, Madison, WI, USA; July 15, 2020: Matthew Campbell, Trinity College Dublin, Dublin, Ireland; July 22, 2020: Krzysztof Kucharz, University of Copenhagen, Copenhagen, Denmark; July 29, 2020: William Elmquist, University of Minnesota, Minneapolis, MN, USA; August 5, 2020: Edward Neuwelt, Oregon Health & Sciences University, OR, USA; August 12, 2020: Robert Thorne, Denali Therapeutics, San Francisco, CA, USA; August 19, 2020: Teresa Sanchez, Weill Cornell Medical College, New York City, NJ, USA; August 26, 2020: Michael Taylor, University of Wisconsin, Madison, MI, USA; September 2, 2020: Joan Abbott, King’s College London, London, UK.

Speakers were offered a training session by the organizers to cover key aspects of a Zoom-based webinar and to test video, audio, and slide presentation prior to the live seminar. Ten out of 16 speakers took the training session. Speakers were informed by the organizers that any data presented should be considered public as security on the Zoom platform could not be ensured. Invited speakers had the opportunity to give permission to have their seminar recorded or to opt out of the recording. If a speaker gave permission to record, the entire seminar was recorded, and recordings were made available to all registered participants in a non-downloadable format through the University of Kentucky Zoom cloud for up to 48 h after the live session. Out of 16 speakers, 15 gave permission to record the live seminar.

### Advertisement and participants

To reach scientists in the brain barriers community, the seminar was advertised by email to participants of previous in-person meetings, and on social media platforms such as Facebook and LinkedIn on personal profiles. In addition, the introductory flyer was kindly distributed via email by the International Brain Barriers Society (IBBS) using their email distribution list. Interested scientists were asked to register by sending their name, affiliation, and career stage to a dedicated email address created for the organization and execution of BBV2020 (bbv2020seminar@gmail.com). Once registered, participants were added to a BBV2020 listserv provided by the University of Kentucky and received weekly updates. These weekly updates included a flyer with the information regarding the upcoming speakers and the specific zoom link for the seminar. Participants also received a follow up email after each seminar with a zoom link to the video recordings. With the permission of the speaker of the week, registered viewers had access to recordings for up to 48 h after the live seminar.

### BBV2020 seminar presentation

Each seminar was started with opening remarks by one of the organizers (Drs. Bauer, Hartz, or Kim) followed by an introduction of the speaker by the moderator. Moderators (Drs. Hudson, Nehra, Pizzo, Storck) rotated each week, only one moderator was assigned to each week. After the seminar presentation, the moderator facilitated the question-and-answer session. Participants were encouraged to ask a live question by raising their digital hand. When a participant raised the digital hand, the moderator would unmute the participant at which point the participant was able to ask the speaker a question directly. In addition, participants had the opportunity to ask questions via the Zoom Q&A or the chat function. Written questions were then subsequently read by the moderator. The moderator concluded each seminar by thanking the speaker and the audience for attending and by announcing the speaker for the following week.

### Map generation

World maps highlighting countries participating in the various meetings and representation were generated using MapChart (www.mapchart.net; license: https://creativecommons.org/licenses/by-sa/4.0/).

### Survey

Toward the end of the seminar series, a survey was generated using Qualtrics (Qualtrics) and run through the University of Alabama Qualtrics system. A link to the survey was sent to all registered seminar participants by email. Questions covered general audience participation, career stage, as well as affective factors. Survey results are only reported in an aggregate.

### Data collection, analysis, and statistics

Survey data was collected through the University of Alabama Qualtrics system and was determined by the University of Alabama IRB to be “*non-human subject research*”. Data from the live webinars were collected by Zoom for each seminar and reported as unique viewers to eliminate counting individuals double who potentially logged on twice. Data on video views were collected from the University of Kentucky Zoom Webinar cloud server and were reported as total accessed views. Survey data from Qualtrics was analyzed with the IBM^®^ SPSS^®^ software platform (IBM, Armonk, NY, USA), using one-way ANOVAs and *t*-tests to identify significant trends [5]. Data with a *p* < 0.05 are considered significant.

## Results

We organized the virtual BBV2020 seminar series for the brain barriers research field to fill the void that the COVID-19 pandemic left due to canceled and postponed meetings in 2020. The seminar series was held between May–September 2020 every Wednesday from 12:00 to 1:00 PM Eastern Daylight Time (EDT). Seminars were hosted on a Zoom webinar platform and featured a 45-min presentation from an invited speaker followed by questions and answers from the audience. In the following sections, we summarize data collected by the Zoom software, from the listserv used, and the participant survey. We compare and contrast the data from the virtual BBV2020 seminar series with data from in-person meetings from past years.

### BBV2020 attendance

**Fig. 1:**
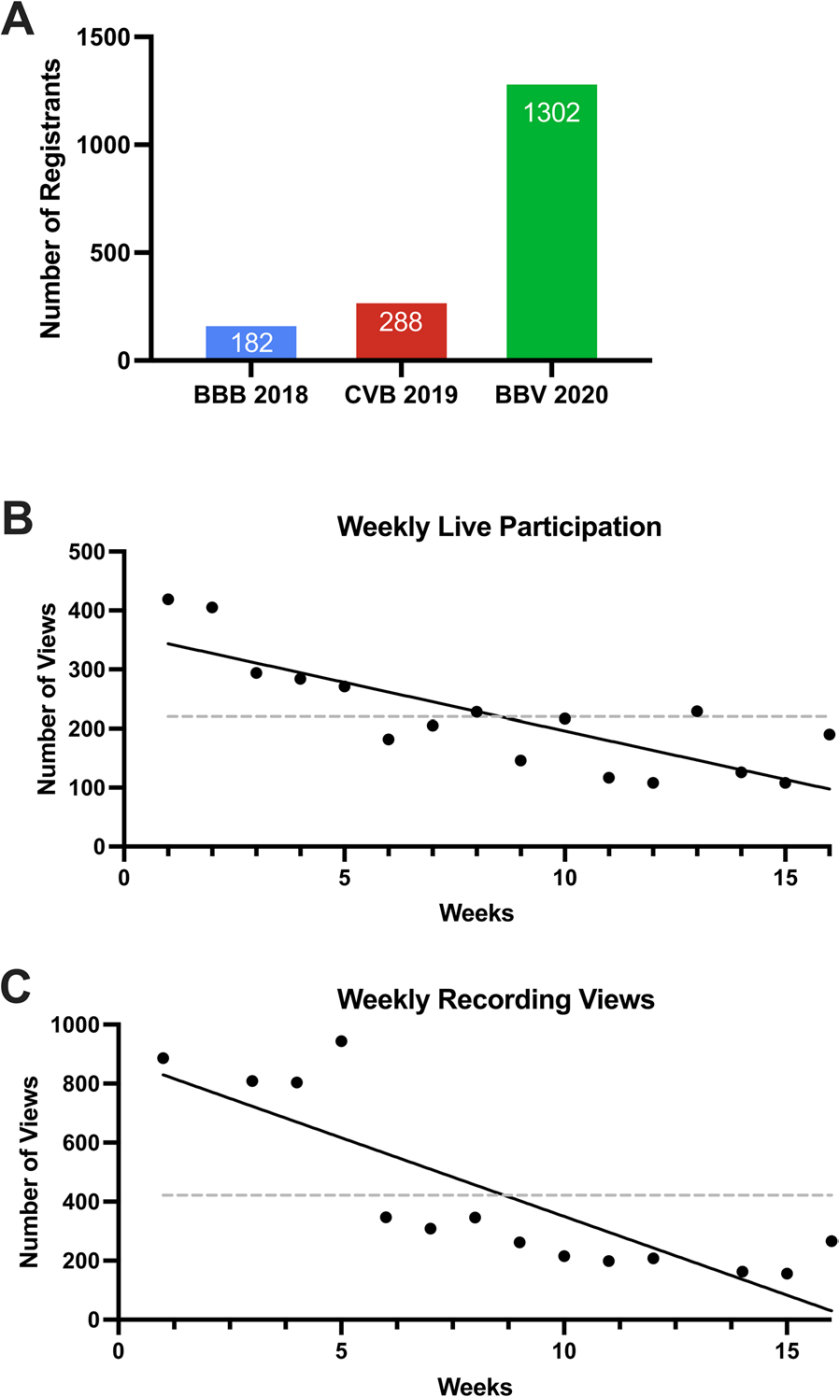
Attendance Trends for Brain Barrier Research Conferences. **A** With 1,302 individuals signing up and added to the listserv, BBV2020 attracted more registrants than BBB 2018 and CVB 2019. **B** Number of unique viewers weekly at the live Zoom Webinar sessions of BBV2020. **C** Number of views (clicks) of the video recordings when available weekly for BBV2020. Black line indicates best linear fit. Gray line indicates the average (mean) for all 16 weeks

On May 14, 2020, we sent out the first advertisement for the BBV2020 seminar series via email using a listserv with email addresses of past conference attendees and via postings using LinkedIn, Facebook, and Twitter. By the start of the first seminar on May 20, 2020, more than 720 scientists had registered. By the end of the 16-week long BBV2020 seminar series, a total of 1302 registrants signed up on our listserv to receive the seminar link, speaker updates, and video recording links (Fig. 1A). We obtained data from two past in-person meetings from the respective conference chairs: (1) brain barriers meeting in 2018 (BBB 2018) and the (2) Cerebral Vascular Biology meeting in 2019 (CVB 2019).

Strikingly, the total number of registered applicants for the BBV2020 was more than 4-fold higher compared to the total number of registered participants for the 2018 and the CVB 2019 meetings (Fig. 1A). The large number of registrants is likely due to free registration, no associated travel expenses, a world-wide reach, and that virtual meetings come with no obligations for the attendees. In contrast, the number of attendees of the in-person meetings (BBB 2018 and CVB2019) was limited and determined by the size of the conference site.

The first live BBV2020 seminar was attended by 419 participants. As the seminar series progressed, the participant number during live seminars gradually decreased, the average participant number was 220, the minimum was 108 (Fig. 1B). A similar trend was observed in the numbers of total recording views: the first seminar recording had a maximum of 944 views; in average, videos had 422 views (Fig. 1C). The trendline in Fig. 1C shows a drop in recording views over the course of the seminar series. These trends held regardless of academic rank as graduate students, postdoctoral fellows, and professors all showed a similar downward trend in live attendance (Additional file 1: Fig. S1A–C). Note that we were unable to capture recording data for week 2 due to a recording error and for week 13 due to the request by the speaker to not record.

Together, the high number of registrants suggests that the virtual BBV2020 seminar series sparked interest. Attendance of the live seminars and the numbers of views for the recorded videos decreased over time, which may be an indicator of “*virtual meeting fatigue*” or attendees being on their summer vacation.

### BBV2020 survey analysis

By the end of the seminar series in September, every BBV2020 registrant received an invitation to participate in an online survey created by the organizers on August 23, 2020. The survey was designed for registrants to provide feedback and consisted of 10 questions: (1) demographic question to capture career level and career type; (2) single select multiple choice question to capture attendance of past in-person brain barrier meetings, (3) single select multiple choice question on how many recorded seminars were viewed, (4) Likert scale question regarding the enrichment BBV2020 provided for the community during the COVID-19 pandemic, (5) dichotomous question asking if the registrant would still participate in BBV2020 seminars if they were continued, (6) single select multiple choice question on attendance frequency, (7) single select multiple choice question on how many times the registrant has thus far attended a brain barriers meeting previously in person, (8) matrix question to capture affective factors, (9) matrix question to capture learning and teaching, and (10) matrix question to capture combined validity and practicality of the BBV2020 series. In addition, registrants responding to the survey had the opportunity to include general comments to the organizers in a separate text box.

**Fig. 2:**
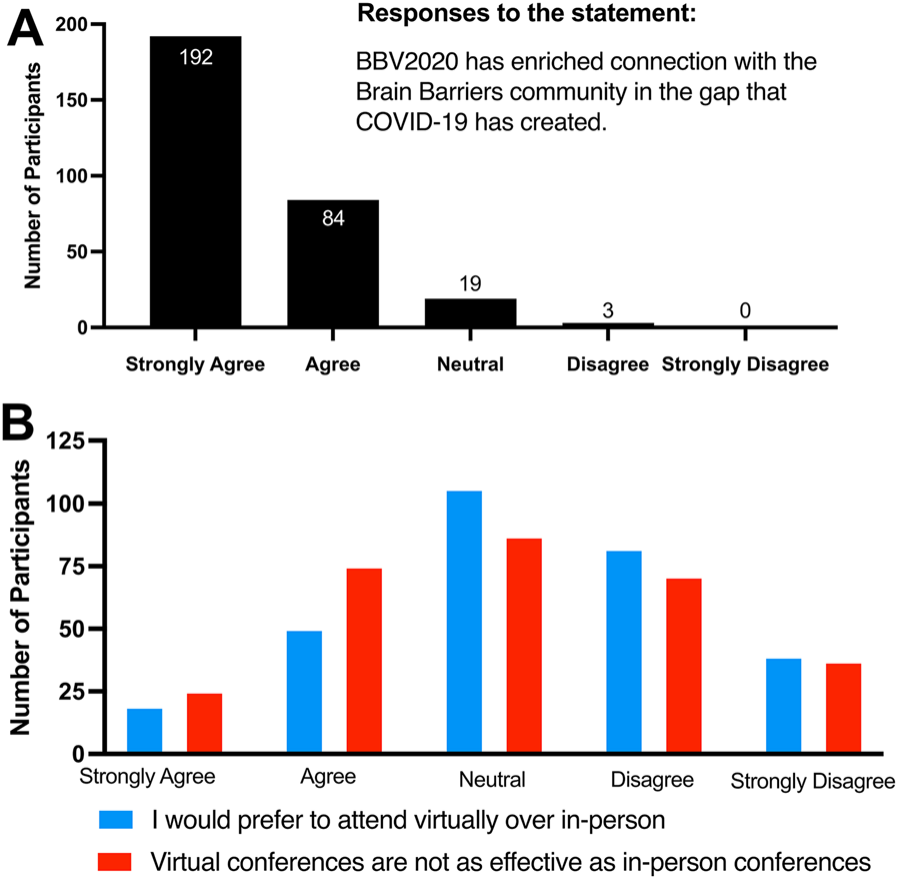
Post-seminar survey results. **A** Volunteer respondent result when posed with the prompt “BBV2020 has enriched connection with the Brain Barriers community in the gap that COVID-19 has created”. **B** Volunteer respondent results when posted with the prompts “In the future, I would prefer to attend a virtual conference over an in-person conference” (Blue) and “The virtual experience is not as effective compared to the in-person conferences” (Red)

Overall, 23% of registrants (total of 299 registrants) responded to the survey. Over 89% of survey respondents agreed strongly that “Attending BBV2020 was a positive experience” and 63% of responders felt that “BBV2020 is appropriate for my research”. Generally, the respondents tended to agree with the following statement: “BBV2020 has enriched the connection with the Brain Barriers community in the gap that COVID-19 has created” (n = 298 respondents to this question of a total of 299 registrants, M = 1.44, SD = 0.660, 1 = Strongly Agree, 2 = Agree, 3 = Neutral, 4 = Disagree, 5 = Strongly Disagree; Fig. 2A). However, when posed with the statements “In the future, I would prefer to attend a virtual conference over an in-person conference” or “The virtual experience is not as effective compared to the in-person conferences”, responses were neutral (M = 3.24; Fig. 2B) indicating a positive view towards a virtual conference. A difference emerged when we compared BBV2020 participants based on their career level with their preference for in-person versus virtual meeting formats: using one-way ANOVA, we found a significant difference between groups (F(9286) = 2.607, *p* = 0.007). Using Tukey’s HSD post hoc test indicated that Master’s students were more likely to disagree with the following statement when compared to Faculty/Professors: “I would feel more comfortable attending Brain Barrier conferences/seminars in person, but was glad to attend virtually due to COVID19.” A strong trend emerged when Master’s and Ph.D. students were grouped together and compared to Post-Docs and Faculty/Professors in an independent-samples *t* test. Results show graduate students in general were more favorable to a virtual conference compared to colleagues more established in their careers (t(225) = -1.972, *p* = 0.061). The lack of significance is in part due to the low sample size (n = 74) for graduate students.

Overall, descriptive data revealed many participants felt BBV2020 was a positive experience. In fact, 99.3% of survey respondents noted they somewhat or strongly agree that their experience was positive, with only 4.7% saying they faced technical difficulties. Additionally, most respondents agreed that virtual conferences have an important role in research (93.2%) and are more accessible than in-person conferences (90.2%). Finally, more than half of respondents (56.9%) noted virtual conferences can do things in-person conferences cannot.

Nevertheless, there continued to be a desire among many BBV2020 participants to return to conferencing in person, with 13.5% saying they found it hard to focus in the virtual format. In fact, roughly one-third (33.9%) felt the virtual conference was not as effective as an in-person conference, and only one-quarter (23.3%) said they would prefer virtual conferences to in-person conferences in the future, while 35.8% were neutral.

Together, our findings suggest that while BBV2020 filled a need created by a global pandemic, the enthusiasm for returning to only in-person meetings remained relatively neutral suggesting that virtual platforms may play a critical role in dissemination of research and science in the future. Moreover, the data from this study show a trend between graduate students versus professors in their response with regard to in-person meetings indicating that career stage may influence the perspective of the usefulness of in-person versus virtual platforms.

### Global reach of BBV2020

**Fig. 3:**
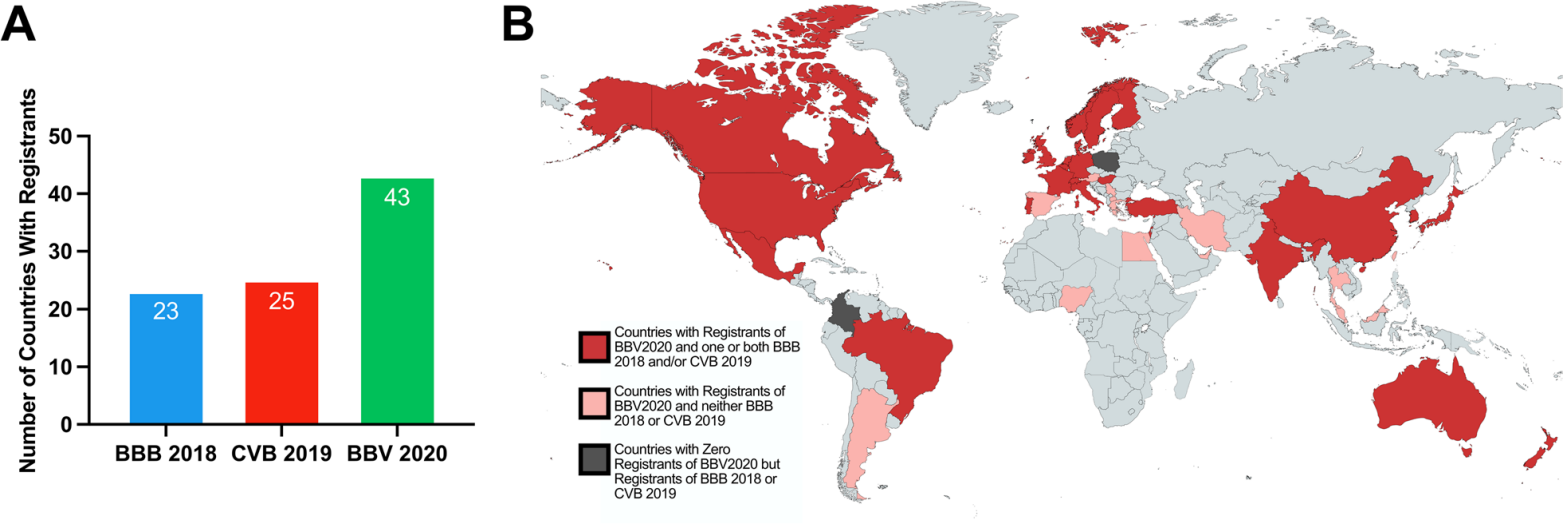
Global reach of BBV2020. **A** BBV2020 had more countries represented compared to BBB 2018 and CVB 2019 alone. **B** World map showing countries represented by BBV2020. Countries where registrants participated in BBV2020: Red and Pink. Countries not represented at BBV2020: Light Gray and Dark Gray. Countries where registrants attended BBV2020 and at least one of the in-person meetings between Meeting 2018 or CVB 2019: Red. Countries where registrants attended BBV2020 and did not attend either BBB 2018 or CVB 2019: Pink. Countries that had no participation in any of the brain barriers meetings examined in this study: Light gray. Countries that had participation in either BBB 2018 or CVB 2019 that did not participate in BBV2020: Dark Gray

During the seminar registration process we collected attendants’ affiliations and based on this information we determined the global reach of the BBV2020. Registrants of the BBV2020 seminar series were from 43 countries representing six of the seven continents (Fig. 3A, B). Compared to the in-person BBB 2018 and the CVB2019, more countries were represented at the BBV2020 (Fig. 3A).

**Fig. 4:**
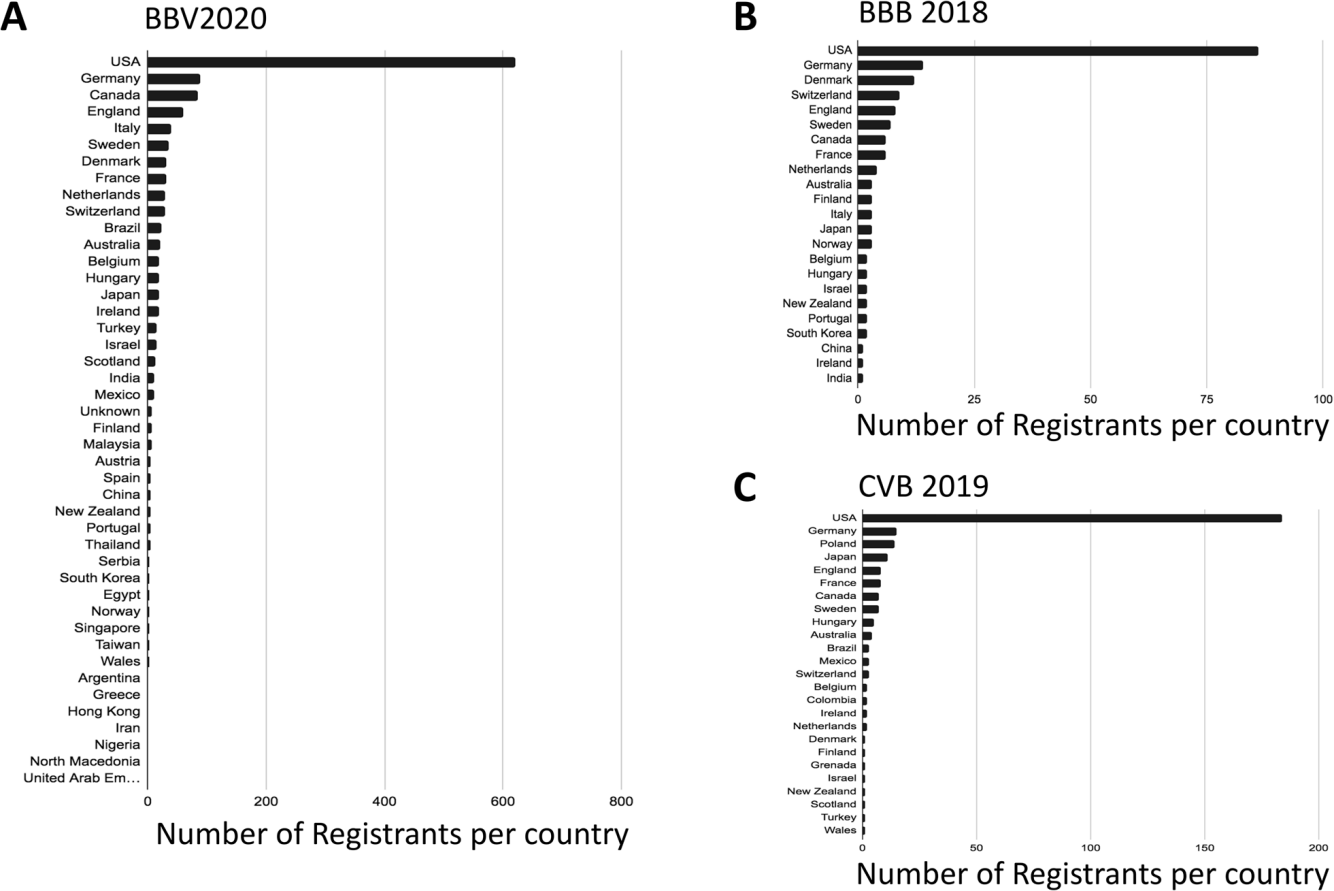

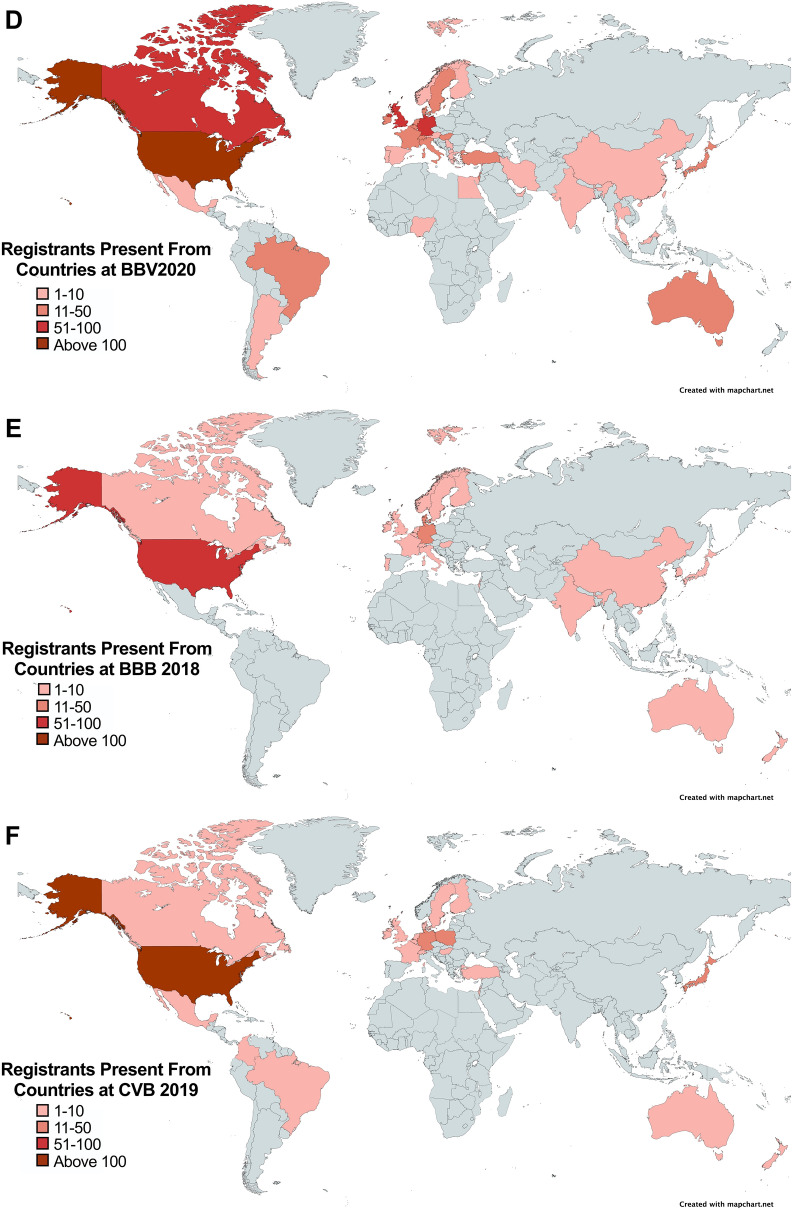
Global view of participation over BBV2020, BBB 2018, and CVB 2019. Representation of participants for (**A**) BBV2020, (**B**) BBB 2018, and (**C**) CVB 2019. Ordered from top to bottom in greatest number of registrants. Number of registrants scaled by color based on number of registrants per country for (**D**) BBV 2020, (**E**) BBB 2018, and (**F**) CVB 2019. Light Pink: 1–10 registrants; Dark Pink: 11–50 registrants; Red: 51–100 registrants and Dark Red: > 100 registrants

We also compared the number of participants from each country who joined BBV2020 vs. BBB 2018 and CVB 2019. We found that the number of participants per country worldwide was also higher compared to both in person meetings (Fig. 4A–F). These data show that the virtual nature of BBV2020 enabled researchers world-wide to participate and suggest that the outreach of a virtual event can extend beyond the field.

### Career stages represented at BBV2020

**Fig. 5:**
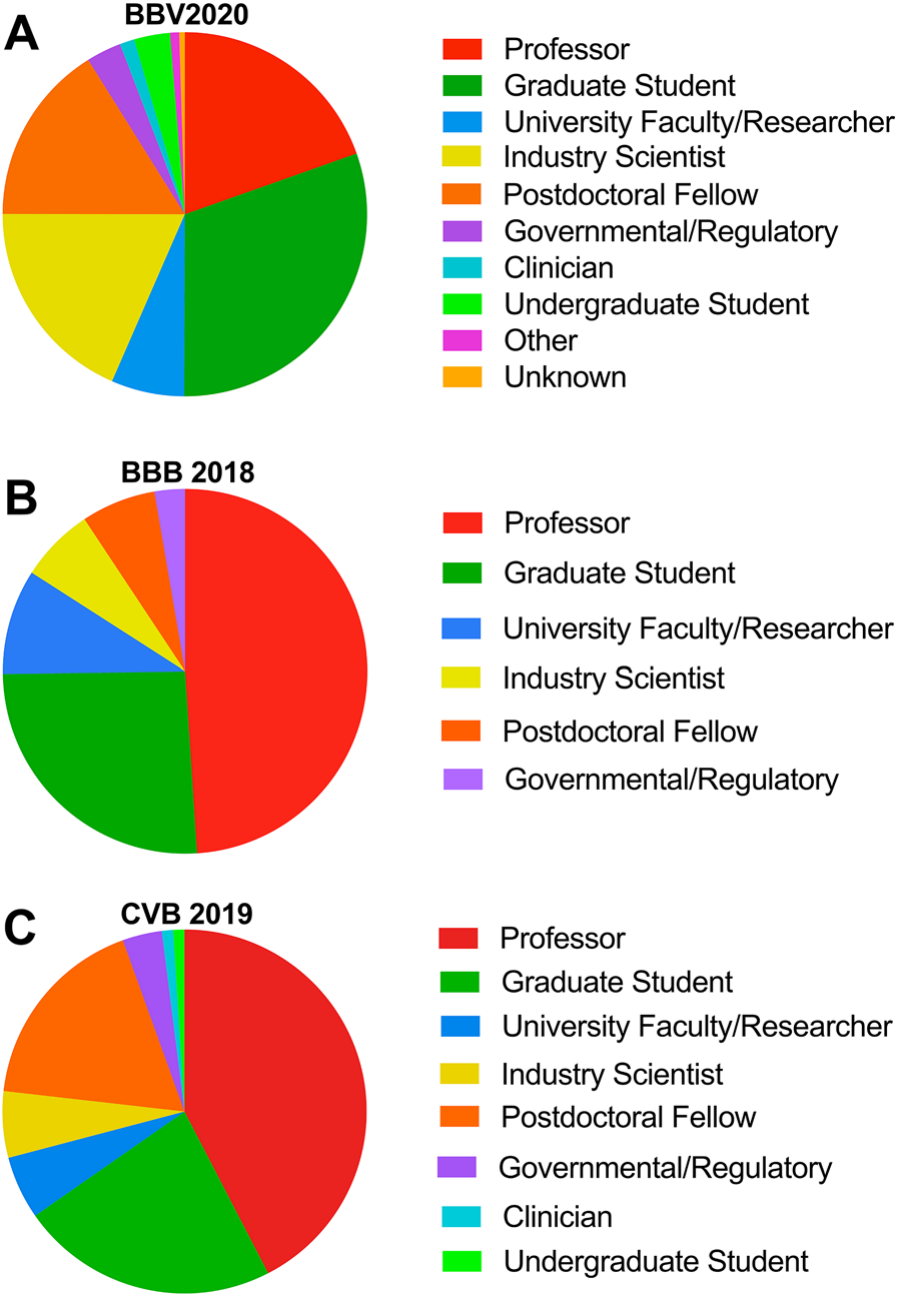
Proportions of various career stages participating in BBV2020, BBB 2018, and CVB 2019. Proportions of various career stages participating in (**A**) BBV2020, (**B**) BBB 2018, and (**C**) CVB 2019. Colors correspond to career stage: Red (professor any rank), Green (graduate student any rank), Dark Blue (research faculty or researcher), Yellow (industry scientist), Orange (postdoctoral fellow), Purple (government/regulatory), Light Blue (clinician), Green (undergraduate student)

We examined the self-reported career stage of BBV2020 participants to that of BBB 2018 and CVB 2019 participants. Based on the survey, more than 46% of all participants attended a brain barriers conference/seminar for the first time. We also found that in-person meetings are attended by 42.4–48.9% academicians at the professor rank, graduate students make up 22.9–25.8% and postdoctoral fellows are 6.6–17.7% of the participants (Fig. 5). In contrast, at the BBV2020 19.6% of academicians were at the professor rank, graduate students made up 30.5% and postdoctoral fellows 16.1% of attendees. We also found that BBV2020 had a larger participation from industry (18.5% vs. 5.6–6.6%), and undergraduate students (3.2% vs. 1%) compared to both in person meetings. Together, our data suggest that a virtual platform seminar like BBV2020 reaches a broader range of scientific ranks and careers.

## Discussion

Seemingly overnight the worldwide COVID-19 pandemic moved virtual meetings, seminars and conferences from the sidelines to center stage. For scientists, connecting on a virtual platform to share data and discuss ideas became the new norm. For many professional societies and research networks a virtual format was a viable option to offset the burden of the pandemic [6, 7]. We organized the BBV2020 seminar series for researchers in the brain barriers field to fill the vacuum canceled in-person conferences left in 2020. Using data collected throughout the BBV2020 seminar series from Zoom Webinar live attendance, number of views from posted videos, and a post-seminar survey, we captured the impact of BBV2020 on the brain barriers field. We compared these data with those from past in-person conferences and assessed the reach of the BBV2020 virtual seminar series with in-person meetings. While we acknowledge that comparisons between a seminar series to a conference may have its limitations, this was the first stand-in virtual event for the brain barriers field that may provide insight for future planning. In the following sections we discuss our findings in the context of the existing literature and provide a future perspective on how a virtual platform could impact the brain barriers field beyond the pandemic.

### Attendance: scientists around the globe

Due to the pandemic, the idea and subsequent organization of the BBV2020 occurred in a relatively short time frame of about 4 weeks. This is in stark contrast to in-person meetings that are often scheduled and organized months and years ahead of the actual event. Advertisement of the BBV2020 encompassed personal email, postings on social media, and utilization of the International Brain Barriers Society email distribution list. Continuous advertisement resulted in the registration of over 1300 scientists. The total registrant number cannot be directly compared to that of in-person meetings since those are more complex in nature, have maximum capacity limits, and involve other factors such as travel expenses and time commitment. Similar to other meetings which transitioned to online formats during the pandemic, BBV2020 had a large increase in registrants when compared to previous in-person meetings (~7-fold and 4.5-fold higher compared to 2018 and 2019, respectively; Fig. 1; [8]. Given that previous brain barriers meetings usually have a few hundred participants, the high number of registrants could be an indicator for high interest in the field beyond traditional brain barrier research groups. In contrast, these high registrant numbers could also be due, in part, to BBV2020 being a no-cost event or could be explained by pandemic-related cancellations resulting in relatively event-free calendar of the participants. While there were more than 1,300 registrants for BBV2020, no more than 419 ever attended a live webinar. Additionally, 323 people registered for BBV2020 but never attended a live webinar. They may, however, have participated by watching the video recordings after the live webinar. A downward trend of attendance numbers for live seminars was observed throughout the course of the 16-week series (Fig. 1B). The recorded seminars drew considerable interest initially with a maximum of 944 views in week 4 of the series, but later in the series, video views dropped to about 200 per seminar (Fig. 1C). Reasons for these downward trends remain speculative, but could be due to the summer break, the selected time for the seminar making it difficult for researchers in certain time zones to attend live, zoom fatigue, or increasing responsibilities in re-opened research laboratories or at home. The downward trends we observed was in contrast to a similar virtual seminar series in another field that remained relatively flat over the course of the summer [4]. Given that average attendance of the BBV2020 was 220 per seminar and typical attendance for in-person conferences in the brain barriers field range between 150 and 300 participants (caveat: one of the in-person conferences has a strict 182-person maximum limit), we conclude that a core group of participants attended most sessions.

### Survey: feedback from participants

A survey conducted after the seminar series revealed that most participants felt that BBV2020 enhanced the connection to the brain barriers scientific community during the COVID-19 pandemic (Fig. 2A). Differences emerged between how graduate students and professors viewed BBV2020: graduate students in general were more favorable of the virtual format compared to faculty. This may be partially explained by a lack of funding for graduate student travel or political “red tape” (e.g. visas) for some researchers [9]. Additionally, students may disproportionately adapt more easily and rapidly to technology usage compared to senior colleagues [10]. Clearly, another survey would be needed to determine if a virtual component outside of a pandemic would be positively viewed by researchers in the brain barriers field.

### Virtual seminars: a powerful opportunity

One major advantage of a virtual seminar series such as the BBV2020 is the lack of a travel burden. Taking off the financial and time-commitment associated with in-person meetings has been shown to increase the diversity of attendees [3, 9, 11]. Based on those findings, we anticipated that BBV2020 would have representation from more countries than past in-person meetings. As shown in Fig. 3, registrants from 43 different countries were represented at BBV2020, which is 40% more compared to BBB 2018 (23 countries) and CVB 2019 (25 countries; Fig. 3). In particular, there was high participation from countries in Southeast Asia, Middle East, Africa, and South America that had not been represented at in-person conferences held in 2018 and 2019 (Figs. 3B, 4). Unsurprisingly however, considering countries represented at previous in-person meetings and due to the number of registrants for BBV2020, there was generally a greater participation from each country represented than in 2018 meeting and CVB 2019 [3, 8]. Due to the time of the BBV2020 live sessions (noon to 1 pm Eastern US Time), the majority of participants were from North America, South America, and Europe (Fig. 4). While time zone challenges are common among virtual events, switching between time zones or holding a “*flipped*” session (flipping evening sessions from Europe to the USA and vice versa) could increase participation from Asia and Oceania [12]. However, offering a virtual series that constantly switches the seminar time may be difficult to manage and could be confusing for participants. Another strength of virtual seminars is that they are more accessible and inclusive especially for junior researchers in the field. The in-person meetings in 2018 and 2019 were dominated by academic faculty at any professor rank and graduate students (Fig. 5). In contrast, BBV2020 saw a large reduction in the percentage of participating faculty (over 40% in person to less than 20% for BBV2020) and a larger proportion of graduate students (22–25% in person to over 30% for BBV2020), industry scientists (about 6% in person to 18.5% for BBV2020), and governmental/regulatory personnel who had signed up (Fig. 5). In addition, a weekly virtual seminar series allows scientist to pick-and-choose the sessions they are interested in, a choice that could interfere with the observed trends shown in Fig. 1. Our data further support the trends observed across a variety of disciplines who have shifted their conferences from in-person to online events, revealing that virtual conferences are more flexible, and more inclusive and accessible worldwide, especially for early-career scientists [9]. Virtual seminars also provide an opportunity for the organizers to invite prominent speakers of a field on a relatively small budget. In general, virtual conferences are known to enhance the accessibility for busy professionals. A recently published *Nature* poll indicates that 74% of scientists want virtual meetings to stay after the pandemic (925 poll responses) [13]. Easier accessibility, lower carbon footprint, and lower costs were the driving factors chosen in favor for virtual meetings in this poll. Moreover, virtual seminars provide an easy and affordable opportunity for researchers with disabilities, researchers with high teaching loads, or for scientists with parenting and other responsibilities to stay connected to their respective research community. During the COVID-19 pandemic, virtual seminars took center stage and raised the standard for accessibility, interactions, inclusivity, and equity in science [9]. Virtual events changed how we think about meetings and they constitute a yet underestimated, but powerful tool to share science and connect with each other. By rethinking how meetings are held, various fields have had different degrees of participation depending on the availability of the talks or content versus live sessions. These virtual platforms have the potential to create a “connectivity cascade” reaching more people than traditional meetings that are confined by a single geographical location and time [14]. BBV2020 had an impressive reach worldwide, however, there was still a notable deficit in participants from underdeveloped countries, especially Africa. These discrepancies have been noted in the context of expanding telehealth to African countries that still remain challenging due to monopolization of telecommunication infrastructure, political will, and access to adequate broadband efficiency [15]. Despite the advantages of BBV2020, we recognize that limitations remain, such as one-way delivery of material to participants. Lack of critical face-to-face interactions allowing junior scientists to have meaningful two-way communication with other participants was notably lacking in BBV2020 as seen in other virtual conferences as well [16]. The future of such virtual seminars, meetings, or conferences may lie in their inclusion as part of in-person meetings according to a recently published “best practice” guideline for virtual meetings [17]. For example, a virtual component could be included in plenary sessions, workshops, small group discussions, poster sessions, and/or social events of in-person conferences. BBV2020 represented only one aspect (a seminar held in the form of a webinar) of these common meeting events, but future events could certainly enhance the virtual experience. Regardless, virtual components could be exploited to increase diversity in the brain barriers research field which is consistent with the NINDS strategy for enhancing the diversity of neuroscience researchers [18].

## Conclusion and future perspectives

Overall, the Virtual BBV2020 Seminar Series created during the COVID-19 pandemic was positively received by the brain barriers research community. Key benefits of virtual seminars like the BBV2020 are that they are convenient, affordable, eco-friendly, reach a broad audience, and are a good tool to increase diversity and equity. Thus, incorporating a virtual component during an in-person meeting or offering a virtual counterpart in addition to in-person meetings could help increase the outreach of a small research field like the brain barriers field. Nevertheless, limitations remain such as a lack of interaction between speakers and participants, which is particularly critical for junior researchers. And although BBV2020 expanded the reach beyond past in-person meetings, challenges reaching scientists in underdeveloped countries remain. Together, BBV2020 was created in a time of crisis but has the potential to thrive in the post-pandemic world. Embracing virtual scientific events may allow us to advance the brain barriers research field and have an impact on how we exchange science in the future.

## Supplementary Information

**Additional file 1: Figure S1.** Attendance Trends for BBV2020 by Academic Rank. Number of unique viewers weekly at the live Zoom Webinar sessions of BBV2020 separated by A) Graduate Student, B) Postdoctoral Fellow, and C) Professor (any rank).

## Data Availability

The datasets used and/or analyzed during the current study are available from the corresponding author on reasonable request.

## Abbreviations

BBB 2018: Blood–brain barrier meeting 2018.
CVB 2019: Cerebral Vascular Biology 2019.
BBV2020: Brain Barriers Virtual 2020.
COVID-19: Coronavirus Disease 2019.
IBBS: International Brain Barriers Society.
EDT: Eastern Daylight Time.
SD: Standard Deviation.
